# Supplementary material for: Validation of Pharmacogenomic Interaction Probability (PIP) Scores in Predicting Drug–Gene, Drug–Drug–Gene, and Drug–Gene–Gene Interaction Risks in a Large Patient Population
Source: J Pers Med. 2022 Nov 29;12(12):1972. doi: 10.3390/jpm12121972 (PMC9783707; doi:10.3390/jpm12121972)
Supplement: Supplementary file 1 [file jpm-12-01972-s001.zip › jpm-1996307-supplementary.pdf]

Supplementary Material

Table S1: High-evidence drugs by clinical area

Clinical Area	Drug Name	Clinical Area	Drug Name
Behavioral Health	amitriptyline	Infectious Disease	abacavir
Behavioral Health	amoxapine	Infectious Disease	atazanavir
Behavioral Health	aripiprazole	Infectious Disease	dolutegravir
Behavioral Health	aripiprazole lauroxil	Infectious Disease	efavirenz
Behavioral Health	atomoxetine	Infectious Disease	quinine
Behavioral Health	brexpiprazole	Infectious Disease	sulfamethoxazole/ trimethoprim
Behavioral Health	citalopram	Infectious Disease	voriconazole
Behavioral Health	clomipramine	Miscellaneous	cevimeline
Behavioral Health	clozapine	Miscellaneous	eliglustat
Behavioral Health	desipramine	Neurology	brivaracetam
Behavioral Health	diazepam	Neurology	carbamazepine
Behavioral Health	doxepin	Neurology	clobazam
Behavioral Health	duloxetine	Neurology	deutetrabenazine
Behavioral Health	escitalopram	Neurology	donepezil

Behavioral Health	fluvoxamine	Neurology	fosphenytoin
Behavioral Health	iloperidone	Neurology	modafinil
Behavioral Health	imipramine	Neurology	oxcarbazepine
Behavioral Health	lofexidine	Neurology	phenytoin
Behavioral Health	nortriptyline	Neurology	pimozide
Behavioral Health	paroxetine	Neurology	pitolisant
Behavioral Health	perphenazine	Neurology	siponimod
Behavioral Health	protriptyline	Neurology	tetrabenazine
Behavioral Health	risperidone	Neurology	valbenazine
Behavioral Health	sertraline	Pain Management	carisoprodol
Behavioral Health	thioridazine	Pain Management	celecoxib
Behavioral Health	trimipramine	Pain Management	codeine
Behavioral Health	venlafaxine	Pain Management	flurbiprofen
Behavioral Health	vortioxetine	Pain Management	hydrocodone
Cardiology	atorvastatin	Pain Management	ibuprofen
Cardiology	carvedilol	Pain Management	meloxicam
Cardiology	clopidogrel	Pain Management	methadone
Cardiology	fluvastatin	Pain Management	oliceridine

Cardiology	hydralazine	Pain Management	piroxicam
Cardiology	lovastatin	Pain Management	tramadol
Cardiology	pitavastatin	Reproductive and Sexual Health	flibanserin
Cardiology	pravastatin	Rheumatology	allopurinol
Cardiology	propafenone	Rheumatology	amifampridine
Cardiology	rosuvastatin	Rheumatology	azathioprine
Cardiology	simvastatin	Transplant	tacrolimus
Cardiology	warfarin	Urology	darifenacin
Endocrinology	elagolix	Urology	fesoterodine
Gastroenterology	dexlansoprazole	Urology	mirabegron
Gastroenterology	dronabinol	Urology	tamsulosin
Gastroenterology	esomeprazole	Urology	tolterodine
Gastroenterology	lansoprazole		
Gastroenterology	meclizine		
Gastroenterology	metoclopramide		
Gastroenterology	omeprazole		
Gastroenterology	ondansetron		
Gastroenterology	pantoprazole		

Gastroenterology	rabeprazole		
Gastroenterology	sulfasalazine		
Hematology/Oncology	belinostat		
Hematology/Oncology	capecitabine		
Hematology/Oncology	eltrombopag		
Hematology/Oncology	erdafitinib		
Hematology/Oncology	fluorouracil		
Hematology/Oncology	fluorouracil topical		
Hematology/Oncology	gefitinib		
Hematology/Oncology	irinotecan		
Hematology/Oncology	mercaptopurine		
Hematology/Oncology	nilotinib		
Hematology/Oncology	pazopanib		
Hematology/Oncology	sacituzumab govitecan-hziy		
Hematology/Oncology	tamoxifen		
Hematology/Oncology	thioguanine		
Hematology/Oncology	avatrombopag		
